# Transcriptomic analysis of hepatic responses to testosterone deficiency in miniature pigs fed a high-cholesterol diet

**DOI:** 10.1186/s12864-015-1283-0

**Published:** 2015-02-06

**Authors:** Zhaowei Cai, Xiaoling Jiang, Yongming Pan, Liang Chen, Lifan Zhang, Keyan Zhu, Yueqin Cai, Yun Ling, Fangming Chen, Xiaoping Xu, Minli Chen

**Affiliations:** Laboratory Animal Research Center, Zhejiang Chinese Medical University, Hangzhou, 310053 China; Department of Cancer Genetics, Roswell Park Cancer Institute, Elm and Carlton Streets, Buffalo, NY 14263 USA; College of Animal Science, Nanjing Agricultural University, Nanjing, 310058 China

**Keywords:** Testosterone, Nonalcoholic fatty liver disease, Hepatic steatosis, Miniature pigs, RNA-Seq

## Abstract

**Background:**

Recent studies have indicated that low serum testosterone levels are associated with increased risk of developing hepatic steatosis; however, the mechanisms mediating this phenomenon have not been fully elucidated. To gain insight into the role of testosterone in modulating hepatic steatosis, we investigated the effects of testosterone on the development of hepatic steatosis in pigs fed a high-fat and high-cholesterol (HFC) diet and profiled hepatic gene expression by RNA-Seq in HFC-fed intact male pigs (IM), castrated male pigs (CM), and castrated male pigs with testosterone replacement (CMT).

**Results:**

Serum testosterone levels were significantly decreased in CM pigs, and testosterone replacement attenuated castration-induced testosterone deficiency. CM pigs showed increased liver injury accompanied by increased hepatocellular steatosis, inflammation, and elevated serum alanine aminotransferase levels compared with IM pigs. Moreover, serum levels of total cholesterol, low-density lipoprotein cholesterol, and triglycerides were markedly increased in CM pigs. Testosterone replacement decreased serum and hepatic lipid levels and improved liver injury in CM pigs. Compared to IM and CMT pigs, CM pigs had lower serum levels of superoxide dismutase but higher levels of malondialdehyde. Gene expression analysis revealed that upregulated genes in the livers of CM pigs were mainly enriched for genes mediating immune and inflammatory responses, oxidative stress, and apoptosis. Surprisingly, the downregulated genes mainly included those that regulate metabolism-related processes, including fatty acid oxidation, steroid biosynthesis, cholesterol and bile acid metabolism, and glucose metabolism. KEGG analysis showed that metabolic pathways, fatty acid degradation, pyruvate metabolism, the tricarboxylic acid cycle, and the nuclear factor-kappaB signaling pathway were the major pathways altered in CM pigs.

**Conclusions:**

This study demonstrated that testosterone deficiency aggravated hypercholesterolemia and hepatic steatosis in pigs fed an HFC diet and that these effects could be reversed by testosterone replacement therapy. Impaired metabolic processes, enhanced immune and inflammatory responses, oxidative stress, and apoptosis may contribute to the increased hepatic steatosis induced by testosterone deficiency and an HFC diet. These results deepened our understanding of the molecular mechanisms of testosterone deficiency-induced hepatic steatosis and provided a foundation for future investigations.

**Electronic supplementary material:**

The online version of this article (doi:10.1186/s12864-015-1283-0) contains supplementary material, which is available to authorized users.

## Background

Serum testosterone levels decline gradually and progressively with aging in men. Low levels of testosterone are associated with metabolic disorders, including obesity, dyslipidemia, hypertension, and insulin resistance [1], all of which contribute to the development of nonalcoholic fatty liver disease (NAFLD) [2]. Recent studies have shown that men with low serum testosterone levels have a higher risk of developing hepatic steatosis [3-5]. Moreover, animal studies have demonstrated that the incidence of hepatic steatosis is increased in testosterone-deficient male mice [6,7]. These findings indicate the important role of testosterone in the pathophysiology of NAFLD. However, nutritional parameters, especially a high-cholesterol diet, have been also associated with the development of NAFLD [2,8]. Hypercholesterolemia induced by dietary cholesterol has recently been indicated as a major risk factor for hepatic steatosis [9,10]. In the absence of testosterone, mice develop hypercholesterolemia when fed a diet with no added cholesterol [11]. Hypercholesterolemia and elevated serum cholesterol levels are also observed in hypogonadal men [12,13]. Therefore, these previous findings suggest that testosterone deficiency and testosterone deficiency-induced hypercholesterolemia may aggravate the severity of NAFLD induced by a high-cholesterol diet. However, the mechanisms underlying the effects of testosterone deficiency on the promotion of diet-induced hepatic steatosis remain unclear.

Previous studies have used rodent models to investigate the influence of testosterone on the development of hepatic steatosis. For example, hepatic androgen receptor-knockout male mice receiving a high-fat diet develop insulin resistance and hepatic steatosis [14]. Moreover, dihydrotestosterone suppresses diet-induced hepatic steatosis in castrated male rats [15], and hepatic lipid deposition is elevated in testicular feminized mice (Tfm) compared with intact wild-type littermate controls [7]. Although rodents are commonly used to study hepatic steatosis and have provided valuable insights into the pathogenesis of this disease, the relevance of these models to human disease is limited [16]. As an alternative to rodent models, large animal models, such as pigs, may be more relevant to the study of NAFLD because these models more closely mimic human physiology and anatomy [16-18]. Importantly, castration-induced sex hormone deficiency in male pigs results in obesity, elevated serum triglycerides (TGs), and increased cholesterol levels [19,20], some of the typical causes of NAFLD, suggesting that pigs are suitable models for studying the effects of sex hormones on NAFLD development.

Despite the importance of testosterone in the regulation of hepatic steatosis, the genomic mechanisms through which testosterone deficiency aggravates diet-induced hepatic steatosis are still unclear. Transcriptome profiling is an effective and widely used tool to identify critical genes and pathways involved in pathological processes. Sequencing-based and hybridization-based approaches represent the two major approaches used in transcriptomics studies [21]. High-throughput RNA sequencing (RNA-Seq) has recently become an attractive method of choice in the study of transcriptomes, providing several advantages over microarrays.

In this study, we sought to gain insights into the roles of testosterone in modulating hepatic steatosis using a porcine model of testosterone deficiency and diet-induced hepatic steatosis. Additionally, we examined the contribution of liver genes to the development of hepatic steatosis induced by testosterone deficiency and a high-cholesterol diet using RNA-Seq. To the best of our knowledge, this is the first description of differences in whole-transcriptome patterns occurring in the liver following castration in pigs fed a high-cholesterol diet.

## Results

### Body weights and serum testosterone levels

**Figure 1:**
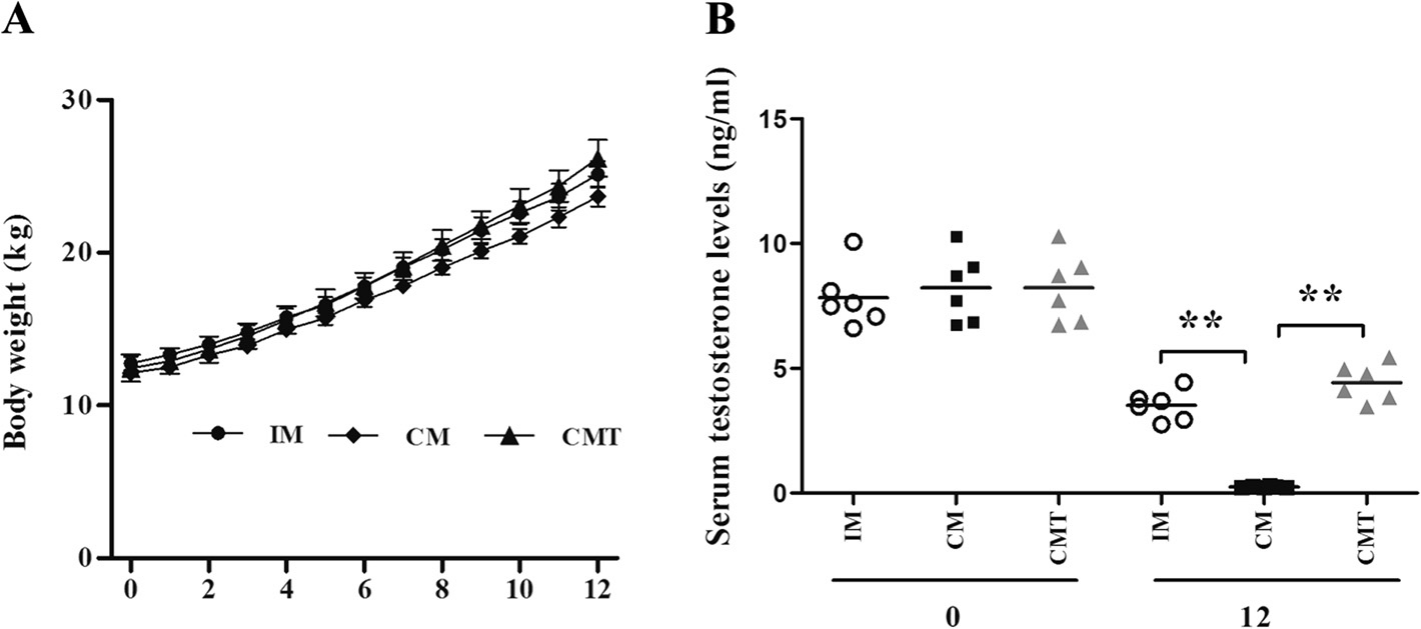
**Effects of castration and testosterone treatment on body weights and serum testosterone concentrations. (A)** Body weights. **(B)** Serum testosterone concentrations. IM: intact male pigs fed an HFC diet; CM: castrated male pigs fed an HFC diet; CMT: castrated male pigs fed an HFC diet and given testosterone replacement therapy. Data are expressed as means ± SEMs, n = 6 per group. **P < 0.01.

Pigs were separated into three groups: intact male pigs fed a high-fat and high-cholesterol (HFC) diet (IM); castrated male pigs fed an HFC diet (CM); and castrated male pigs fed an HFC diet and given testosterone replacement therapy (CMT). The body weights of pigs in each group were calculated and found to be linearly elevated over time. Initial body weights were similar in pigs from the IM, CM, and CMT groups (12.76 ± 0.45, 12.14 ± 0.35, and 12.35 ± 0.89 kg, respectively). Castrated pigs fed an HFC diet gained less weight than did pigs in the other groups. With testosterone replacement, the body weights of CMT pigs increased and were similar to the body weights of pigs in the IM group (Figure 1A).

Serum testosterone concentrations were not significantly different between groups before the study began (0 weeks). As expected, castration caused a significant decrease in serum testosterone levels. The concentration of serum testosterone at 12 weeks was significantly lower in CM pigs than in IM pigs (0.24 ± 0.05 vs. 3.52 ± 0.25 ng/mL, respectively). Testosterone treatment increased serum testosterone levels to 4.43 ± 0.31 ng/mL (Figure 1B).

### Liver weight, body fat content, and serum biochemical parameters

Liver weight, body fat content, and serum biochemical parameters after 12 weeks of HFC feeding are presented in Table 1. The liver weights in CM pigs were higher than those observed in IM pigs, but lower than those in CMT pigs (*P* > 0.05). Liver weight indexes in CM pigs were higher than those in IM and CMT pigs, but these differences were not significant (*P* > 0.05). CM pigs had higher body fat percentages than IM (*P* < 0.01) and CMT pigs (*P* < 0.05). Serum alanine aminotransferase (ALT) levels were significantly increased in CM pigs compared with IM + HFC pigs (*P* < 0.05) and significantly reduced in CMT pigs by testosterone replacement (*P* > 0.05). Aspartate aminotransferase (AST) levels were highest in CM pigs, but the differences were not significant among groups (*P* > 0.05). Compared with IM and CMT pigs, CM pigs showed obviously lower serum levels of superoxide dismutase (SOD, *P* < 0.05) but higher levels of malondialdehyde (MDA, *P* < 0.01). Serum free fatty acid (FFA) levels tended to be higher in CM pigs than in IM and CMT pigs. Fasting serum glucose and insulin levels and homeostatic model assessment of insulin resistance (HOMA-IR) were not significantly different between CM and IM pigs (*P* > 0.05). However, testosterone replacement significant reduced serum insulin levels and HOMA-IR in CM pigs (*P* < 0.01).

**Table 1:** **Liver weight, body fat content and serum biochemical parameters in miniature pigs fed a high-fat and high-cholesterol diet**

	**IM**	**CM**	**CMT**
Body weight (kg)	25.15 ± 0.86	23.69 ± 0.64	26.21 ± 1.20
Liver weight (g)	483.82 ± 47.04	524.72 ± 69.88	558.38 ± 27.95
Liver weight index (%)	19.18 ± 1.74	22.01 ± 2.47	21.30 ± 0.39
Body fat (%)	0.15 ± 0.00^**^	0.24 ± 0.02	0.19 ± 0.01^#^
Serum			
ALT (IU/L)	37.17 ± 2.20^*^	46.88 ± 0.86	42.45 ± 3.68
AST (IU/L)	42.37 ± 4.95	44.70 ± 6.86	41.45 ± 2.60
SOD (U/mL)	88.61 ± 7.09^*^	52.07 ± 9.58	100.69 ± 11.79^##^
GSH-PX (μmol/L)	810.07 ± 65.53	723.44 ± 61.82	900.43 ± 58.22
MDA(nmol/L)	3.30 ± 0.32^**^	5.89 ± 0.68	2.81 ± 0.19^##^
FFA (mmol/L)	0.70 ± 0.13	0.98 ± 0.14	0.65 ± 0.10
Glucose (mmol/L)	5.09 ± 0.13	4.94 ± 0.14	5.15 ± 0.15
Insulin (mIU/L)	27.40 ± 0.66	28.60 ± 0.76	21.1 ± 0.47^##^
HOMA-IR	6.17 ± 0.10	6.29 ± 0.33	4.81 ± 0.35^##^

*P < 0.05 and **P < 0.01, IM vs. CM; ^#^ *P* < 0.05 and ^##^ *P* < 0.01, CMT vs. CM.

### Serum lipids

**Figure 2:**
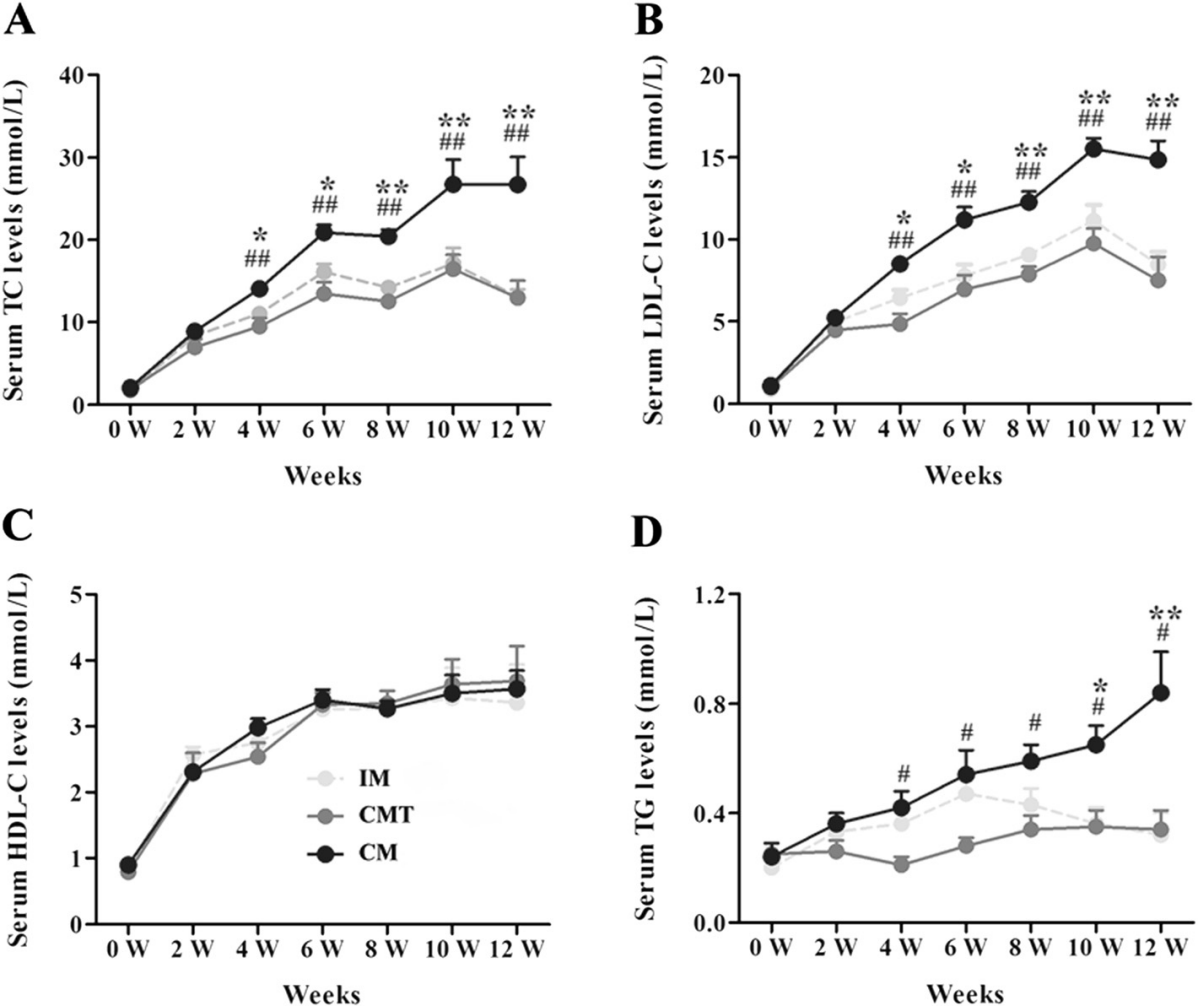
**Effects of castration and testosterone treatment on serum lipid levels. (A)** Serum total cholesterol (TC) levels. **(B)** Serum low-density lipoprotein cholesterol (LDL-C) levels. **(C)** Serum high-density lipoprotein cholesterol (HDL-C) levels. **(D)** Serum triglyceride (TG) levels. IM: intact male pigs fed an HFC diet; CM: castrated male pigs fed an HFC diet; CMT: castrated male pigs fed an HFC diet and given testosterone replacement therapy. Data are expressed as means ± SEMs, n = 6 per group. *P < 0.05 and **P < 0.01, IM vs. CM; ^#^ *P* < 0.05 and ^##^ *P* < 0.01, CMT vs. CM.

To analyze the effects of castration and testosterone treatment on lipid metabolism in HFC-fed pigs, serum lipids were measured at 0, 2, 4, 6, 8, 10, and 12 weeks postcastration. Serum total cholesterol (TC), high-density lipoprotein cholesterol (HDL-C), low-density lipoprotein cholesterol (LDL-C), and TGs were significantly increased in miniature pigs fed an HFC diet (Figure 2). CM pigs had higher TC levels than did IM and CMT pigs from the fourth week after HFC supplementation (*P* < 0.05; Figure 2A). There were no significant differences in serum TC levels between IM and CMT pigs (*P* > 0.05). Serum LDL-C followed a pattern similar to that of TC in all groups of pigs (Figure 2C). However, testosterone deficiency caused by castration had no significant influence on serum HDL-C levels in HFC-fed pigs (Figure 2B). Serum TG levels in CM pigs were higher than those in IM pigs at weeks 10 and 12 (*P* < 0.05) and those in CMT pigs beginning at week 4 (*P* < 0.05; Figure 2D).

### Liver histopathology and hepatic lipids

**Figure 3:**
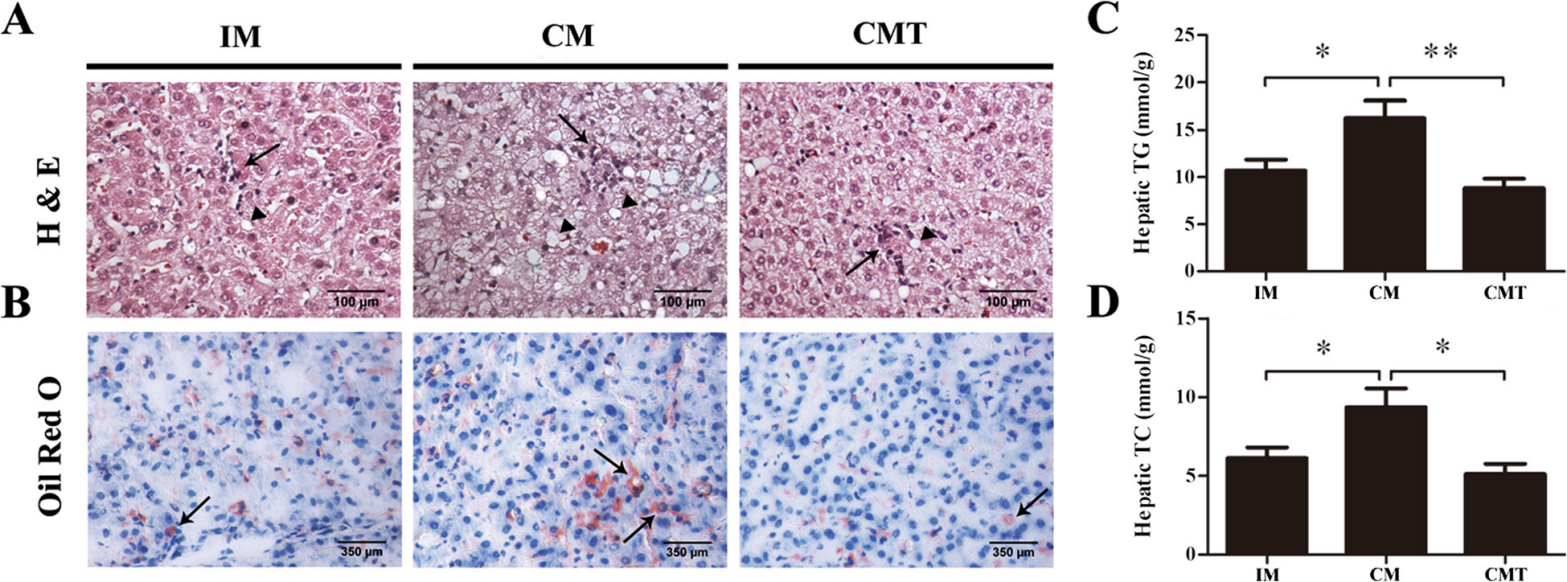
**Effects of testosterone on liver histology and hepatic lipids. (A)** Liver sections stained with hematoxylin and eosin. Micro- and macrovesicular steatosis are indicated by arrow heads, and inflammation is indicated by arrows. **(B)** Oil red O staining of fat in hepatocytes. Increased lipid deposition is indicated by arrows. **(C)** Hepatic triglyceride (TG) content in IM, CM, and CMT pigs. **(D)** Hepatic cholesterol (TC) content in IM, CM, and CMT pigs. IM: intact male pigs fed an HFC diet; CM: castrated male pigs fed an HFC diet; CMT: castrated male pigs fed an HFC diet and given testosterone replacement therapy. Data are expressed as means ± SEMs, n = 6 per group. *P < 0.05; **P < 0.01.

Liver histology results showed that testosterone deficiency induced by castration significantly increased hepatic fat accumulation in pigs fed an HFC diet (Figure 3). CM pigs developed massive micro- or macrovesicular steatosis, while the hepatocytes of IM pigs showed only mild microvesicular steatosis (Figure 3A). Moreover, castration significantly aggravated hepatic inflammation in pigs fed an HFC diet (Figure 3A). Testosterone treatment clearly improved hepatic steatosis and reduced hepatic necroinflammation in CM pigs (Figure 3A). Oil red O staining confirmed the occurrence of increased lipid deposition in the livers of CM pigs (Figure 3B).

Consistent with the observed increase in lipid deposition, we also detected marked increases in TG contents in the livers of CM pigs (Figure 3C). Moreover, testosterone treatment reduced hepatic TG contents in CM pigs (Figure 3C). Likewise, hepatic cholesterol contents in CM pigs were also significantly higher than those in IM and CMT pigs (*P* < 0.05; Figure 3D).

### Gene expression analysis by RNA-Seq

To obtain a global view of the hepatic transcriptome responses to testosterone deficiency and testosterone treatment in pigs fed an HFC diet, we performed comparative RNA-Seq analyses of the liver transcriptomes. cDNA libraries were constructed using total RNA isolated from the livers of IM, CM, and CMT pigs. Using an Illumina HiSeq 2000 sequencer, we obtained approximately 40.58, 45.39, and 32.33 million high quality clean reads from IM, CM, and CMT pigs, respectively. For each sample, ~87% of reads could be mapped to the pig reference genome; of these mapped reads, ~79% aligned with unique genes unambiguously (Table 2).

**Table 2:** **Summary of the sequencing reads alignment to the reference genome**

**Statistics term**	**IM**	**CM**	**CMT**
All reads	40578646	45391430	32329976
Unmapped reads	4949040	5702066	4154466
Mapped reads	35629606	39689364	28175510
Mapping rate	0.878	0.874	0.871
Unique mapping	32288705	36243263	25183689
Unique mapping rate	0.796	0.798	0.779
Repeat mapping	3340701	3445877	2991640

**Figure 4:**
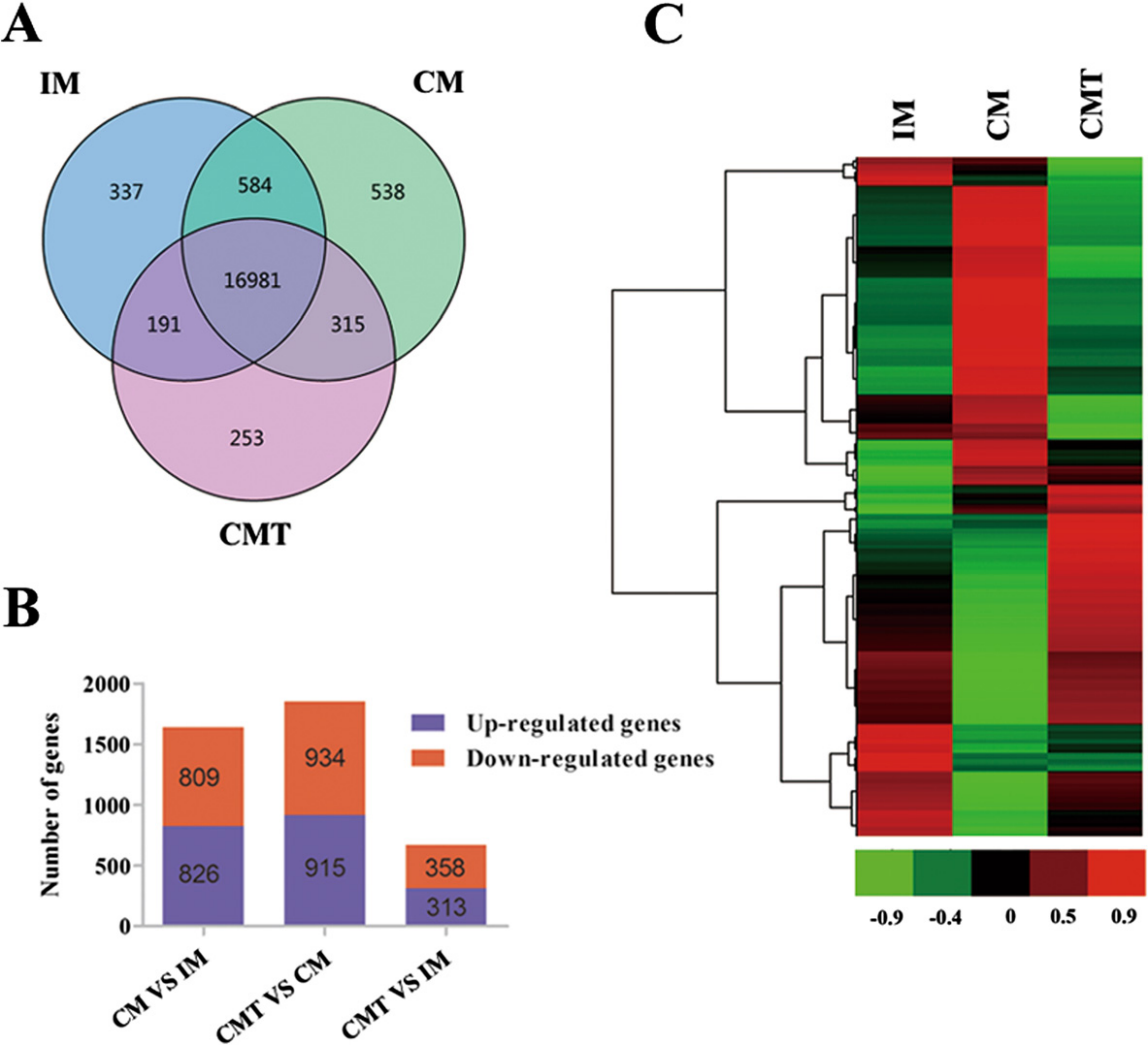
**RNA-Seq analyses of differentially expressed genes (DEGs) in livers of IM, CM, and CMT pigs. (A)** The numbers of upregulated and downregulated DEGs between groups. **(B)** Venn diagram showing the overlap of DEGs between groups. **(C)** Heat map for hierarchical cluster analysis of DEGs between samples. Red: upregulated genes; yellow: downregulated genes. IM: intact male pigs fed an HFC diet; CM: castrated male pigs fed an HFC diet; CMT: castrated male pigs fed an HFC diet and given testosterone replacement therapy.

In total, 18093, 18418, and 17740 expressed genes were detected in the livers of IM, CM, and CMT pigs, respectively. Of these genes, a total of 16981 genes were expressed in all three groups; 584, 315, and 191 genes were identified commonly between each pair of groups (CM versus IM, CMT versus CM, and CMT versus IM, respectively), while 337, 538, and 253 genes were discovered exclusively for IM, CM, and CMT, respectively (Additional file 1; Figure 4A). To identify differentially expressed genes (DEGs) in the livers of IM, CM, and CMT pigs, gene expression data from each group were compared using DEGSeq software [22]. Significance scores were corrected for multiple testing using the Benjamini-Hochberg correction [23]. We used the following criteria to identify DEGs: (1) gene expression level greater than or equal to 1 fragment per kilobases of exon per million fragments mapped (FPKM) in all samples; (2) change in expression level greater than or equal to 1.5 fold; and (3) a false discovery rate (FDR) of less than 0.05 [24]. Thus, a total of 1635 (826 upregulated and 809 downregulated), 1847 (915 upregulated and 934 downregulated), and 671 (313 upregulated and 358 downregulated) DEGs were detected in the livers of pigs for CM versus IM, CMT versus CM, and CMT versus IM groups, respectively (Figure 4B). In total, 2595 different genes were differentially expressed between any two groups (Figure 4C; DEG list provided in Additional file 2).

### Gene Ontology (GO) enrichment analysis of DEGs

To gain further insights into the biological functions of DEGs, we performed GO analyses by querying each DEG identified in the livers of IM, CM, and CMT pigs against the GO database. GO analysis of significantly upregulated DEGs between CM and IM pigs revealed genes that were mainly enriched in immune-related processes, such as the type I interferon signaling pathway, the cytokine-mediated signaling pathway, immune responses, innate immune responses, antigen processing and presentation, and apoptosis (Additional file 3). Downregulated DEGs were mainly involved in metabolism-related processes, such as lipid metabolism, fatty acid metabolism, fatty acid beta-oxidation, bile acid metabolic processes, steroid metabolic processes, ketone body biosynthetic processes, cholesterol metabolic processes, and the tricarboxylic acid and gluconeogenesis pathways (Additional file 3). Upregulated DEGs between CMT and CM pigs revealed genes that were mainly involved in metabolism-related processes, such as lipid metabolism, fatty acid metabolism, fatty acid beta-oxidation, steroid metabolism, bile acid metabolism, cholesterol metabolism, ketone body biosynthesis, and the tricarboxylic acid and gluconeogenesis cycles, while downregulated DEGs were mainly enriched in immune-related processes, such as the type I interferon signaling pathway, the cytokine-mediated signaling pathway, innate immune responses, immune responses, apoptosis, antigen processing and presentation, inflammatory responses, and responses to oxidative stress (Additional file 4).

**Figure 5:**
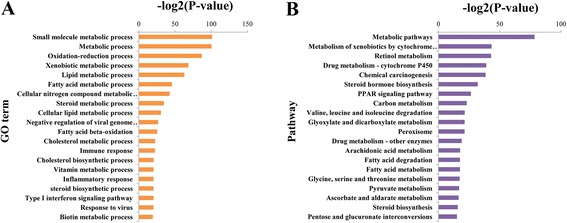
**Gene ontology (GO) and pathway analysis of differentially expressed genes (DEGs). (A)** Top 20 significant GO terms (biological processes) associated with the identified DEGs. The vertical axis represents the GO category, and the horizontal axis represents the -Log2(*P*-value) of the significant GO terms. **(B)** Top 20 significant pathways involving DEGs. The vertical axis represents the pathway category, and the horizontal axis represents the -Log2(*P*-value) of the significant pathways. Greater -Log2(*P*-value) scores correlated with increased statistical significance.

The enriched GO analyses of all significant DEGs between any two groups were also investigated and were found to be mainly involved in small molecule metabolism, metabolic processes, lipid metabolism, fatty acid metabolism, steroid metabolism, cholesterol metabolism, fatty acid beta-oxidation, immune responses, inflammatory responses, apoptosis, and responses to oxidative stress (Figure 5A, Additional file 5).

### Pathway analysis of DEGs

Pathway analysis using the KEGG database was performed to determine significant pathways involving the DEGs identified in this study. Our results showed that 64 pathways were significantly enriched for the identified DEGs (*P* < 0.05; Figure 5B). The enriched pathways are listed in Additional file 6. Moreover, pathway analysis showed that these genes were mainly involved in metabolic pathways, steroid hormone biosynthesis, the PPAR signaling pathway, peroxisomes, fatty acid degradation, pyruvate metabolism, steroid biosynthesis, the nuclear factor-kappaB (NF-κB) signaling pathway, primary bile acid biosynthesis, antigen processing and presentation, the tricarboxylic acid cycle, synthesis and degradation of ketone bodies, and glycerolipid metabolism (Figure 5B, Additional file 6).

### Series-cluster analysis of DEGs

**Figure 6:**
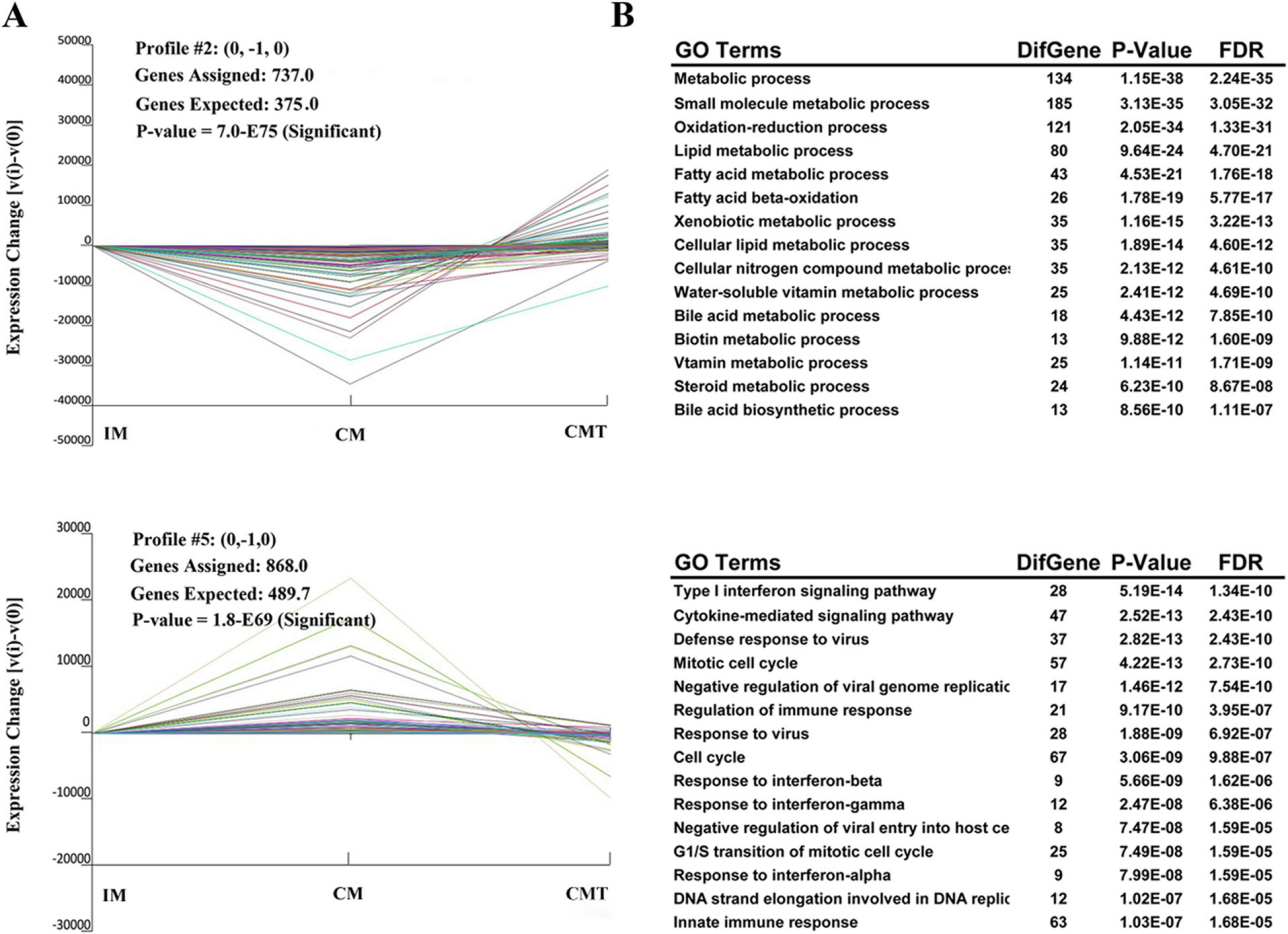
**Significant gene expression tendencies and gene ontology (GO) analysis. (A)** Differentially expressed genes between the groups described in Additional file 3 (Table S4) were separated into seven possible model profiles, including two significant gene expression tendencies (profiles 2 and 5). Profile 2 included 737 genes that were decreased in CM pigs, but increased in CMT pigs. Profile 5 included 838 genes that were increased in CM pigs, but decreased in CMT pigs. **(B)** Significantly enriched GO terms in profiles 2 and 5. The top 15 biological functions and the case genes in each cluster are listed. IM: intact male pigs fed an HFC diet; CM: castrated male pigs fed an HFC diet; CMT: castrated male pigs fed an HFC diet and given testosterone replacement therapy.

To refine the set of genes that were differentially expressed between any group, we categorized the 2595 DEGs into seven possible model profiles (Additional file 7) to enrich for gene expression tendencies using the Short Time-Series Expression Miner (STEM) program [25]. We identified two patterns of gene expression (profiles 2 and 5, Additional file 8) with significance (*P* < 0.05; Figure 6A). Profile 2 contained 738 genes that were characterized by decreased expression in CM pigs but increased expression in the CMT group. In contrast, profile 5 contained 869 genes that were characterized by increased expression in CM pigs but exhibited decreased expression in the CMT group. Significantly enriched GO terms from profiles 5 and 2 are illustrated in Figure 6B and Additional files 9 and 10. Cluster analysis showed that the significantly enriched GO terms from profile 5 were closely correlated with immune and inflammatory responses. The included genes were enriched in the type I interferon signaling pathway, cytokine-mediated signaling pathways, regulation of immune responses, innate immune responses, immune responses, and antigen processing and presentation (Figure 6B, Additional file 9). Profile 5 genes were also enriched in apoptotic processes and positive regulation of chemokine secretion (Additional file 9).

**Figure 7:**
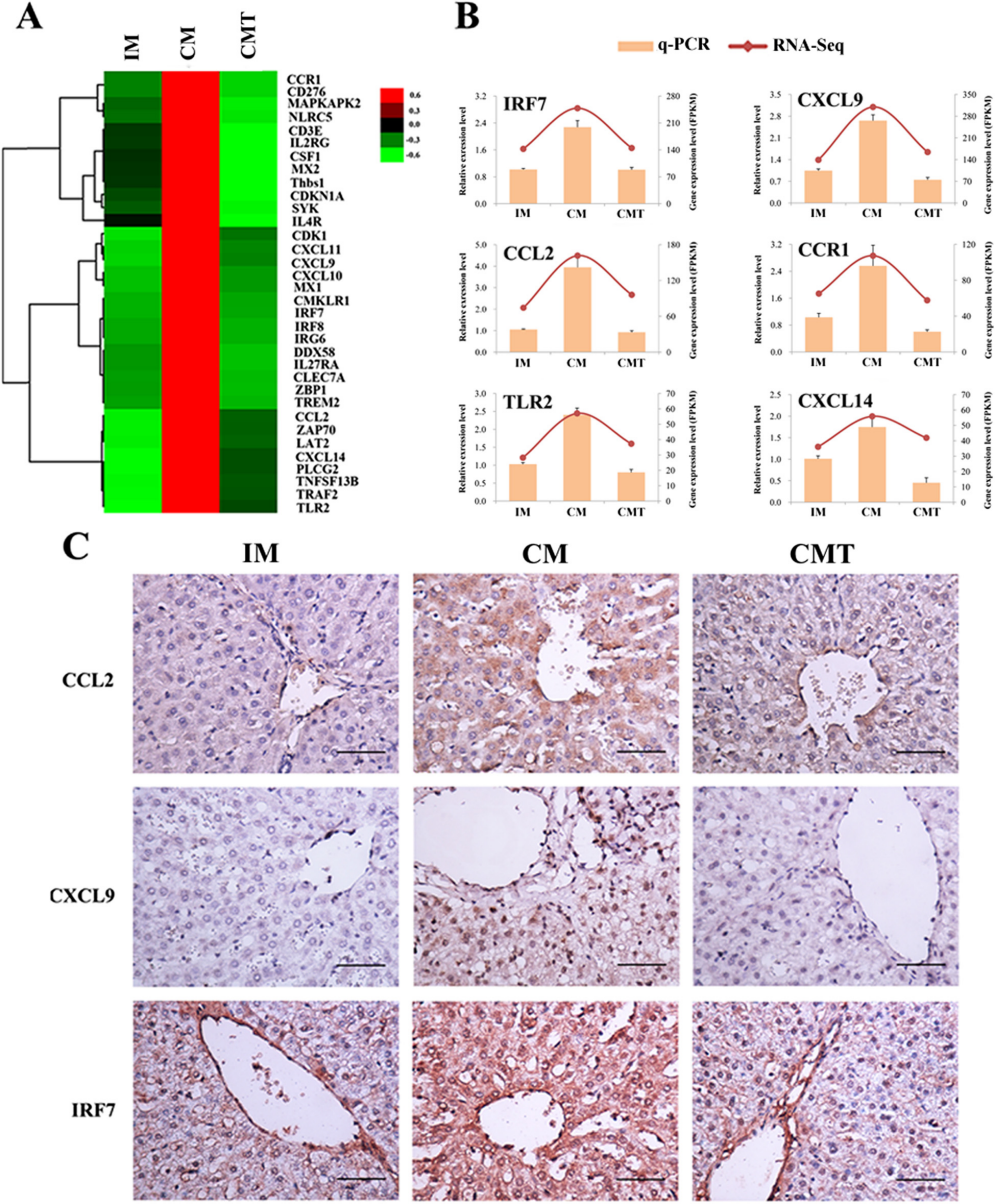
**Expression of genes involved in immune and inflammatory responses. (A)** Heat map for genes involved in immune and inflammatory responses. **(B)** Expression levels of six genes detected by quantitative real-time RT-PCR (yellow box) were in agreement with the RNA-Seq results. Data are expressed as means ± SEMs, n = 6 per group. **(C)** Immunohistochemical assessment of chemokine (C-C motif) ligand 2 (CCL2), chemokine (C-X-C motif) ligand 9 (CXCL9), and interferon-regulatory factor 7 (IRF7), showing expression changes that were in accordance with the RNA-Seq and qRT-PCR results. IM: intact male pigs fed an HFC diet; CM: castrated male pigs fed an HFC diet; CMT: castrated male pigs fed an HFC diet and given testosterone replacement therapy. Scale bars = 100 μm.

The genes involved in immune and inflammatory responses are displayed and clustered in Figure 7A. To verify the observed differential mRNA expression of immune and inflammatory response genes, we performed quantitative real-time RT-PCR (qRT-PCR) using RNA samples extracted from the livers of IM, CM, and CMT pigs. The hepatic expression of six immune and inflammatory response-related genes (*IRF7*, *CXCL9*, *CXCL14*, *CCL2*, *CCR1*, and *TLR-2*) was analyzed in the three groups. Consistent with RNA-Seq results, the expression of these six genes was upregulated in CM pigs and downregulated in CMT pigs after testosterone treatment (Figure 7B). In addition to qRT-PCR validation experiments, we also employed immunohistochemistry to examine the protein expression levels of CCL2, CXCL9, and IRF7 (Figure 7C), which are involved in the development of NAFLD. Based on immunostaining results, we observed similar differences in expression at the protein level. Staining levels tended to be increased in the livers of CM pigs compared to those observed in IM pigs (Figure 7C). However, this positive staining was reduced after testosterone treatment (Figure 7C).

**Figure 8:**
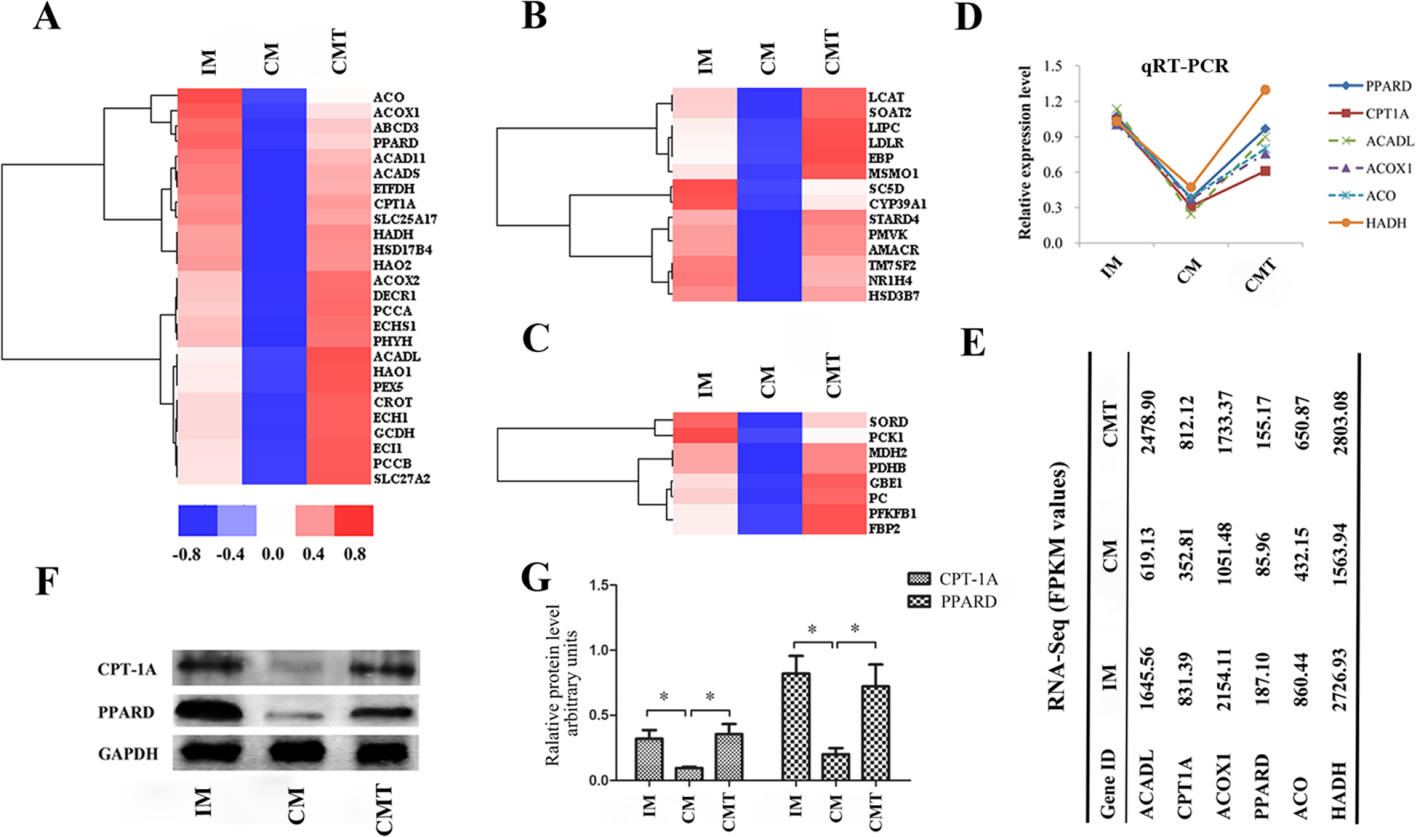
**Expression of genes involved in fatty acid oxidation and cholesterol, bile acid, and glucose metabolism. (A)** Heat map for genes involved in fatty acid oxidation. **(B)** Heat map for genes involved in cholesterol and bile acid metabolism. **(C)** Heat map for genes involved in glucose metabolism. **(D, E)** Expression levels of six genes detected by quantitative real-time RT-PCR (qRT-PCR) that were in agreement with the RNA-Seq results. Data are expressed as means ± SEMs, n = 6 per group. **(F, G)** Western blot analysis of peroxisome proliferator-activated receptor delta (PPARD) and carnitine palmitoyltransferase 1A (CPT-1A) showing expression changes that were in accordance with RNA-Seq and qRT-PCR results. IM: intact male pigs fed an HFC diet; CM: castrated male pigs fed an HFC diet; CMT: castrated male pigs fed an HFC diet and given testosterone replacement therapy. Data are expressed as means ± SEMs, n = 5 per group. *P < 0.05.

Profile 2 genes were mainly enriched in various metabolic subprocesses, including fatty acid metabolism, fatty acid beta-oxidation, steroid biosynthesis, cholesterol and bile acid metabolism, and glucose metabolism (Figure 6B, Additional file 10). We found that the most genes enriched in fatty acid metabolic processes were involved in fatty acid oxidation (Figure 8A). It is well known that impaired fatty acid oxidation contributes to NAFLD; thus, we analyzed the genes participating in fatty acid oxidation further. Carnitine palmitoyltransferase 1A (*CPT-1A*) encodes the rate-limiting enzyme in fatty acid oxidation, which was downregulated 2.36-fold in the livers of CM pigs and upregulated 2.30-fold in the livers of CMT pigs. Peroxisome proliferator-activated receptor delta (*PPARD*) belongs to a class of ligand-dependent transcription factors involved in hepatic lipid metabolism and was downregulated 2.18-fold in the livers of CM pigs and upregulated 1.81-fold in the livers of CMT pigs. Moreover, the *ACADL*, *ACOX1*, *ACO*, and *ECIl* genes, encoding long-chain acyl-CoA dehydrogenase, palmitoyl acyl-CoA oxidase 1, acyl-CoA oxidase, and mitochondrial enoyl-CoA isomerase, respectively, were 2.66-, 2.05-, 1.99-, and 1.95-fold downregulated in the livers of CM pigs and 4.00-, 1.65-, 1.51-, and 2.52-fold upregulated in the livers of CMT pigs, respectively (Figure 8).

Enriched genes mediating cholesterol, bile acid, and glucose metabolism are also displayed in Figure 8. Genes involved in cholesterol biosynthesis (*PMVK* and *TM7SF2*), cholesterol esterification (*LCAT* and *SORT2*), cholesterol transport and absorption (*LDLR* and *LIPC*), and bile acid metabolic processes (*NR1H4* and *HSD3B7*) were downregulated in the livers of CM pigs, but restored by testosterone treatment (Figure 8B). Several genes encoding enzymes involved in glucose metabolism were also downregulated in CM pigs. For example, *PCK1*, encoding the rate-limiting gluconeogenesis enzyme phosphoenolpyruvate carboxykinase (PEPCK), and *PFKFB1*, encoding the glycolysis enzyme 6-phosphofructo-2-kinase, were decreased in the livers of CM pigs, but increased in the livers of CMT pigs after testosterone treatment (Figure 8C).

We performed qRT-PCR to verify the mRNA expression profiles of the fatty acid oxidation-related genes *CPT1A*, *PPARD*, *ACADL*, *ACO*, *ACOX1*, and 3-hydroxyacyl-CoA dehydrogenase (*HADH*). Consistent with RNA-Seq analysis, the expression of these six genes was downregulated in CM pigs and upregulated in CMT pigs after testosterone treatment (Figure 8D, E). We then measured the protein expression levels of CPT1A and PPARD by western blot analysis. The protein levels of CPT1A and PPARD were decreased in the livers of CM pigs as compared to IM pigs and were increased in CMT pigs after testosterone treatment (Figure 8F, G).

### Pathway-Act-Network analysis

**Figure 9:**
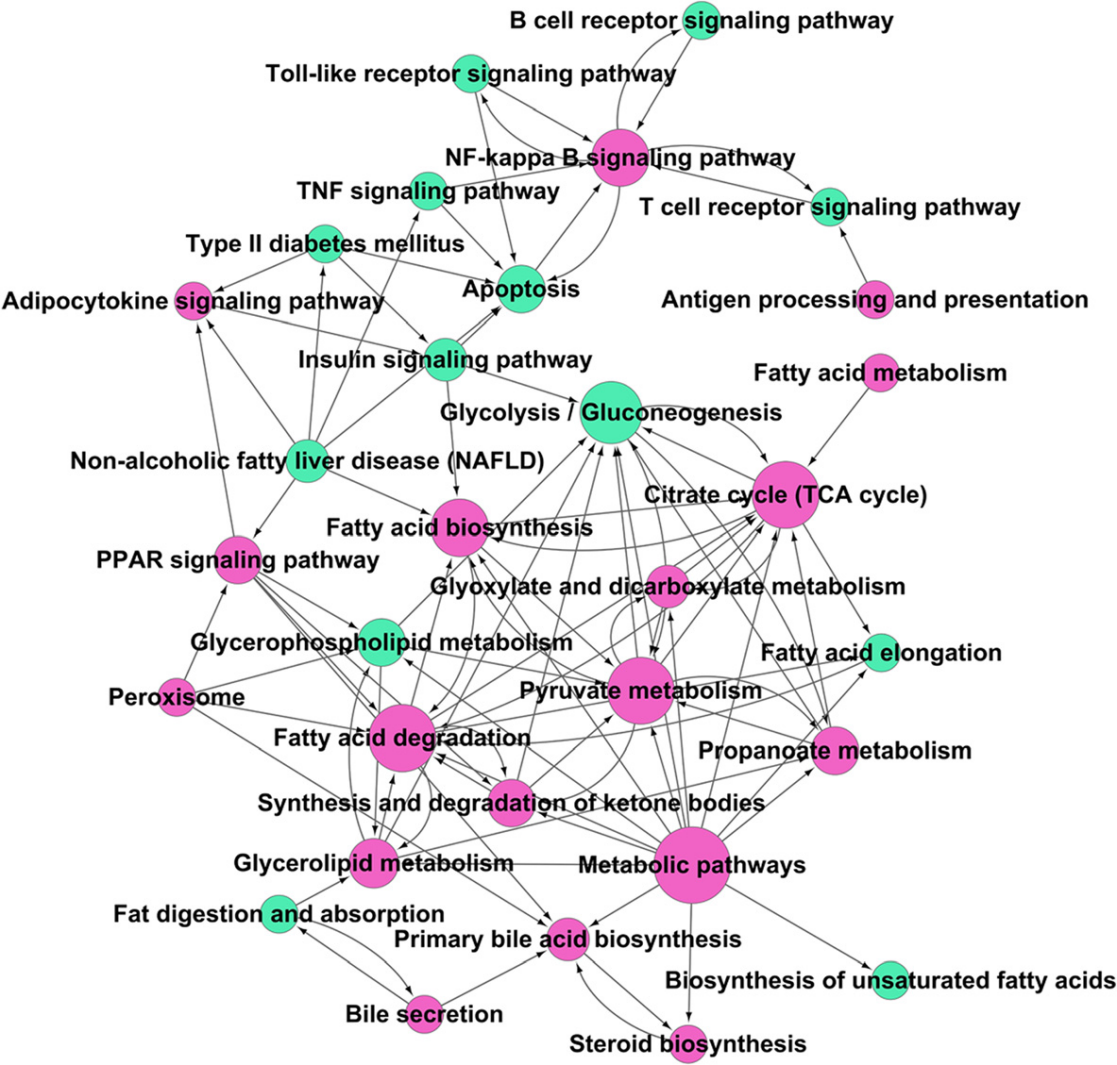
**Pathway-Act-Network analysis.** The Pathway-Act-Network was built according to the interactions with pathways identified in the KEGG database. Cycle nodes represent pathways, and the arrow between two nodes represents an interaction target between pathways. The size of nodes represents the power of the interaction among the pathways (Additional file 11). Red nodes represent significant pathways and green nodes represent nonsignificant pathways.

To further understand the importance of pathway interactions and to screen key pathways for significant roles in NAFLD induced by testosterone deficiency and an HFC diet, we built a Pathway-Act-Network according to the direct or systemic interactions assigned between pathways in the KEGG database (Figure 9, Additional file 11). As shown in Figure 9, some DEGs involved in key pathways during NAFLD were identified, including metabolic pathways, fatty acid degradation, pyruvate metabolism, and the citrate cycle. Moreover, glycerolipid metabolism and the NF-κB signaling pathway in the interaction network were also predicted to play important roles.

To display a detailed pathway of fatty acid degradation and the relative differences in genes influenced by testosterone, a putative pathway of fatty acid degradation was constructed based on KEGG mapping. Enzymes and proteins involved in regulating fatty acid degradation (CPT-1A, ACO, and ACADL) were decreased in CM pigs, but recovered in CMT pigs (Additional file 12). We also constructed a putative pathway of peroxisome-related processes according to KEGG mapping (Additional file 13). Similarly, several genes involved in regulating peroxisome processes, such as *PMP70* and *PEX14*, were decreased in CM pigs, but increased in CMT pigs.

## Discussion

In this work, we studied the effects of testosterone on the development of hepatic steatosis in pigs fed an HFC diet. Our data indicated that serum cholesterol (TC and LDL-C, but not HDL-C) and TG levels were significantly higher in CM pigs than in IM pigs. Moreover, testosterone deficiency exacerbated liver injury and increased hepatic TG contents (hepatic steatosis) in CM pigs. However, testosterone replacement attenuated these effects in CM pigs, suggesting that testosterone may play a protective role in diet-induced hepatic steatosis. In previous studies, researchers have used rodent models to evaluate the effects of testosterone on NAFLD; however, several of these were genetic animal models, i.e., androgen receptor (AR)-knockout mice [14] and Tfm mutant mice [7]. These models may have some utility for specific research aims, but most humans that develop NAFLD have few genetic defects. Other studies have also used orchidectomized mouse models; however, these animals do not have increased adiposity [26] or have reduced serum TG levels [6,26], which are inconsistent with findings in humans. We showed here that a porcine model of NAFLD induced by testosterone deficiency and HFC diet mimicked human fatty liver disease in terms of histological analyses and metabolic profiles. To the best of our knowledge, the present study is the first to demonstrate that testosterone deficiency aggravates diet-induced hepatic steatosis in a porcine model. Testosterone deficiency also caused significant changes in body fat percentages and serum biochemical parameters. Previous studies have shown that enzymes involved in fatty acid synthesis are increased in adipose tissues of castrated male pigs [19]. Thus, the higher body fat content observed in CM pigs may be due to the increased fatty acid synthesis in adipose tissue [27]. Estradiol has been reported to decrease fatty acid synthesis by increasing the phosphorylation of acetyl coenzyme-A carboxylase in rats fed a high-fat diet [15]. Moreover, previous studies have reported that serum ALT levels are significantly elevated in ovariectomized mice fed an HFC diet [8], and estradiol administration reduces hepatic MDA content but increases SOD activity in rats with hepatic fibrosis [28]. Thus, the significant changes in body fat and serum parameters induced by castration may be partly attributed to a reduction in estradiol [20]. However, we did not measure hepatic estradiol levels, and elucidation of the role of estrogens in the regulation of fat metabolism and serum parameters was not the primary goal of our study; these will be investigated in future studies.

In this study, we found that testosterone deficiency did not affect serum fasting glucose and insulin levels or HOMA-IR in CM pigs compared to IM pigs. However, testosterone replacement significantly reduced serum insulin levels and HOMA-IR in CM pigs. While previous studies have shown that androgen deficiency increases fasting glucose levels and reduces insulin sensitivity (IR) [29,30], the effects of testosterone on glucose metabolism and IR in clinical observations and experimental studies have not been consistent. For example, several studies have shown that low serum testosterone levels are associated with IR in men and that androgen therapy improves IR and fasting glucose levels in men [31,32]. However, other studies have found that reduction of testosterone levels in men did not significantly affect fasting glucose levels or IR [33,34]. Moreover, castration results in slightly increased fasting glucose levels, but has no effects on fasting insulin concentrations and IR in rats [35]. However, Nikoleanko et al. [26] showed that fasting glucose, fasting insulin, and HOMA-IR levels were not elevated in castrated male rats compared to intact animals and animals receiving testosterone replacement therapy. Using different mouse strains and diets, Inoue et al. [36] also indicated that castration does not induce serum glucose and insulin levels or related IR markers in any of the mouse models studied. To date, few reports have described the effects of testosterone on glucose metabolism and IR in pigs. Our previous study showed that there were no differences in serum levels of glucose and insulin between intact and castrated male pigs fed a normal diet [19]. In the present study, we still found that testosterone deficiency did not change fasting serum glucose and insulin levels in pigs fed an HFC diet. Our results were not consistent with those obtained by Christofferson et al. [20], who found that castration results in disturbed glucose metabolism. These discrepancies may be explained by breed differences and the different experimental model of testosterone deficiency.

Little is known regarding the molecular mechanisms underlying the testosterone deficiency-mediated promotion of fat deposition in the liver. Hepatic steatosis is regulated by multiple metabolic pathways. Previous studies have shown that disturbances in fatty acid oxidation account for excess lipid storage in the liver [37,38]. We found that many genes involved in fatty acid oxidation were downregulated in the livers of CM pigs, and these effects were prevented by testosterone replacement. PPARD regulates lipid oxidation processes [39,40], but its role in hepatic lipid metabolism remains unclear. We found that the levels of PPARD mRNA and protein were significantly reduced in the livers of CM pigs, and testosterone treatment restored the expression of PPARD in CMT pigs. These results are consistent with those obtained by Barroso et al. [41] and Bolic et al. [42], who showed that PPARD activation stimulates hepatic fatty acid oxidation and alleviates diet-induced hepatic steatosis. Moreover, this observation suggests that PPARD may be regulated by sex hormones and plays key roles in the regulation of hepatic steatosis; this provides a novel view of PPARD function in the modulation of hepatic lipid metabolism. In addition to its effects on lipid metabolism, PPARD has been reported to play roles in the regulation of glucose metabolism and IR [40,43]. Studies in genetic and diet-induced IR or diabetic mice have shown that PPARD exerts beneficial effects to ameliorate IR [40,43-45]. Because of our data regarding PPARD, and since the involvement of PPARD in IR is well established, we would expect that markers of IR in CM pigs should be elevated compared with those in IM and CMT pigs. However, there was no difference in fasting glucose, insulin, or HOMA-IR levels in CM pigs compared to IM pigs. Previous studies have indicated that PPARD regulates glucose flux and IR through the pentose phosphate pathway [40,43,46]. However, our transcriptomics data did not reveal any changes in the expression levels of glucose-6-phosphate dehydrogenase (G-6-PDH) and phosphogluconate dehydrogenase (PGD) (two key enzymes in the pentose phosphate pathway) in the livers of CM pigs compared to those of IM pigs. Moreover, PPARD has been reported to reduce glucose production indirectly via activation of AMPK rather than through a direct mechanism [40,47]. In our study, expression levels of AMPK and adiponectin signaling genes (ADIPOQ, ADIPOR1, and ADIPOR2) were not changed in CM pigs compared to IM pigs. Activation of PPARD has failed to enhance severe IR [47-49]. Moreover, in recent studies in rats and mice fed a high-fat diet, heterogeneous effects of PPARD on IR have been observed [49]. Thus, the reasons for the discrepancy between PPARD expression and IR markers in this study may be due to the different effects of PPARD on IR in different species [49]. However, testosterone replacement significantly improved fasting insulin and HOMA-IR levels and restored PPARD expression in CM pigs, despite the observation that gonadectomy apparently did not change fasting insulin, glucose, or HOMA-IR levels compared to those in intact pigs. CPT-1A is the rate-limiting enzyme of mitochondrial β-oxidation [50]. We found that hepatic CPT1A mRNA and protein levels were significantly reduced in the livers of CM pigs and subsequently restored in CMT pigs receiving testosterone supplementation. Other fatty acid oxidation-related genes, such as *ACADL*, *ACOX1*, *ACO*, *HADH*, and short chain enoyl-CoA hydratase (*ECHS1*) were also downregulated in the livers of CM pigs. Taken together, our results provided strong evidence that testosterone deficiency aggravated hepatic fat accumulation in CM pigs partly due to decreased hepatic fatty acid oxidation. Our results are consistent with a human study in which men with short-term hypogonadism showed decreased whole-body lipid oxidation [51]. Moreover, Lin et al. [14] observed reduced fatty acid oxidation in isolated primary hepatocytes from hepatic AR-knockout (H-AR^-/y^) mice, consistent with our results. Peroxisomes have many important functions in lipid metabolism, including fatty acid β-oxidation. A previous study reported that impaired peroxisome function contributes to NAFLD in mice [52]. In this study, we also found that the *PMP70*, *PEX14*, and *PEM34* genes, which are involved in peroxisome-related processes, were downregulated in the livers of CM pigs. These results suggested that testosterone deficiency led to increased hepatic steatosis in CM pigs by affecting peroxisome function.

In addition to the decrease in liver fat oxidation, increased fat synthesis may also contribute to elevated liver fat accumulation [53]. TGs are the main lipids that accumulate during hepatic steatosis [54]. Glycerol 3-phosphate acyltransferase (GPAT) and diacylglycerol acyltransferase (DGAT) catalyze the initial and final steps in TG synthesis, respectively [55,56]. Increased levels of *DGAT1* mRNA occur in the livers of humans with NAFLD [57], indicating the importance of DGAT1 in fatty liver development. Mice lacking DGAT1 have reduced tissue TG levels and are protected against hepatic steatosis [56]. Studies using GPAT-knockout mice [58] and mice overexpressing GPAT in the liver [55] have implicated this enzyme in hepatic steatosis. Stearoyl-CoA desaturase (SCD) catalyzes the de novo biosynthesis of monounsaturated fatty acids (mainly oleate and palmitoleate), which are critical substrates for the synthesis of TGs by GPAT [59]. To date, few studies have been conducted to explore the effects of testosterone on hepatic TG synthesis enzymes. Senmaru et al. [6] reported that orchidectomized mice fed HFD showed significantly increased DGAT2 expression. A recent study revealed that SCD1 expression was significantly elevated in Tfm mice than in wild-type controls [7]. These observations are consistent with our findings that the expression of the TG synthesis genes *GPAT*, *DAGT*, and *SCD* were significantly upregulated in the livers of CM pigs and that testosterone replacement reduced their expression in CM pigs. Our results suggested that testosterone deficiency might increase liver fat accumulation by inducing hepatic TG synthesis. Acetyl-CoA carboxylase alpha (ACACA) and fatty acid synthase (FASN) are also key lipogenic enzymes involved in hepatic lipid deposition. A recent study showed that hepatic ACACA and FASN expression levels were elevated in Tfm mice compared with wide-type littermates [7]. However, Nikolaenko et al. [26] found that there were no significant differences in liver FASN protein levels in C + HFD mice after castration. These results are inconsistent with our findings, which showed decreased ACACA and FASN expression in the livers of CM pigs. These differences might be species-specific or result from different experimental models of hepatic steatosis. In addition, hepatic fatty acid synthesis requires acetyl-CoA generated from multiple metabolic pathways, including glycolysis, the tricarboxylic acid cycle, and fatty acid β-oxidation [60]. Thus, the observed decrease in ACACA and FASN expression may be due to impaired glucose metabolism or fatty acid oxidation in the present study.

The spectrum of NAFLD ranges from simple fatty liver (hepatic steatosis) to nonalcoholic steatohepatitis (NASH; steatosis with inflammation and fibrosis). The “two-hit” theory of NAFLD progression proposes that inflammation, oxidative stress, and apoptosis play critical roles in the pathological progression of NAFLD [61]. Gene expression profiling indicated that several immune and inflammatory response genes were activated in the livers of CM pigs. Chemokines direct the trafficking of immune cells to sites of inflammation [62]. Chemokine (C-C motif) ligand 2 (CCL2; commonly known as monocyte chemoattractant protein-1 [MCP-1]) is one of the most important chemokines involved in inflammation, and elevated levels of CCL2 have been confirmed in the livers of patients with NAFLD [63,64]. Moreover, increased hepatic and serum MCP-1 expression levels have been described in diet-induced NAFLD [62,65]. Expression levels of C-C chemokine receptor 1 (CCR1) and chemokine (C-X-C motif) ligands 9, 10, 14 (CXCL9, CXCL10, and CXCL14) are increased in livers during HFD-induced hepatic steatosis [65-67]. We found that testosterone deficiency significantly increased mRNA and protein levels of the CCL2, CXCL9, CCR1, CXCL10, and CXCL14 chemokines in the livers of CM pigs, and testosterone replacement abolished these effects. Our data suggested that the induction of pro-inflammatory chemokines contributes to the mechanism of inflammatory recruitment in hepatic steatosis induced by testosterone deficiency and an HFC diet. Moreover, clinical and experimental observations have shown that tumor necrosis factor (TNF) may have a pathogenic role during NAFLD development, specifically by modulating chronic lobular inflammation with hepatocellular injury [63,67]. We found that hepatic TNF expression was reduced in CM pigs. This is inconsistent with previous observations [63,68]. Dela Pena et al. [69] demonstrated that knocking out the *TNF* gene does not prevent diet-induced NAFLD in mice. Similar studies by Deng et al. [70] showed that TNFR1-knockout mice still develop hepatic steatosis. Taken together, these studies suggest that TNF may not be a critical mediator of inflammation in this experimental form of hepatic steatosis. In the present study, numerous immunity and inflammation-related processes, including antigen processing and presentation, cytokine-mediated signaling pathways, and chronic inflammatory responses were induced in the livers of CM pigs. Interestingly, previous studies have shown marked induction of numerous immunity- and inflammation-related pathways in PPARD−/− mice [46]. Moreover, activation of PPARD may improve hepatic steatosis via directly suppressing the expression of cytokines or transcription factors associated with inflammation [71,72]. These observations suggest that testosterone deficiency may induce immune and inflammatory responses through modulation of hepatic PPARD expression. Moreover, testosterone has been suggested to act directly on immune cells by repressing transcription factors (such as FOS, JUN, and others) [73,74]. In our study, CM pigs had higher FOS expression than IM pigs. Moreover, testosterone replacement significantly reduced the increased FOS expression in CMT pigs. These results suggested that testosterone may inhibit liver FOS expression in CM pigs, thereby regulating the expression of genes involved in the immune response [74]. Additionally, previous studies have shown that testosterone can bind to intracellular receptors located in immune cells and activate hormone-responsive genes [74,75]. In our study, androgen receptor expression was reduced by about 1.8 fold in the livers of CM pigs compared to those in IM and CMT pigs, suggesting that testosterone may interact with immune cells through its receptor. Therefore, further studies are needed to elucidate the precise mechanisms mediating the observed responses.

Oxidative stress plays critical roles during the development of NAFLD [9]. SOD1 is an important antioxidant enzyme that can reduce reactive oxygen species and protect hepatocytes. SOD1 deficiency caused high levels of oxidative stress in the liver, resulting in hepatic lipid accumulation in mice [76]. In the present study, hepatic *SOD1* gene expression was significantly decreased in the livers of CM pigs compared to IM pigs, suggestive of a reduced defense mechanism against oxidative stress. In contrast to SOD1, cytochrome P450 2E1 (CYP2E1) exerts pro-oxidant activity and may enhance oxidative stress. CYP2E1 expression in the liver is increased in humans and animal models of NAFLD [77]. Moreover, CYP2E1-null mice are protected against HFD-induced obesity and hepatic steatosis [78]. We showed that testosterone deficiency significantly induced hepatic expression of *CYP2E1* and that testosterone replacement abolished these effects. Our data are in accordance with a previous study showing higher levels of CYP enzymes (including CYP2E1) in hepatic tissues of castrated ethanol-fed micropigs than in noncastrated counterparts [79]. Increased oxidative stress may induce hepatocyte apoptosis, resulting in more severe liver injury [80]. Although hepatic apoptosis was not detected histologically, it is important to note that several genes associated with apoptosis, including B-cell leukemia/lymphoma-2 (*BCL2*), caspase-2 (*CASP2*), and bcl-2 associated-x (*BAX*), were upregulated in the livers of CM + HFC pigs in response to testosterone deficiency in the present study. Similar to our study, Nikolaenko et al. [26] demonstrated that testosterone treatment improved diet-induced hepatic apoptosis in mice fed a high-fat diet.

The limitations of the current study include the small number of animals studied and the lack of confirmation of the results using human samples. Despite the fact that pigs have served as a suitable animal model for studying NAFLD, few animal models can entirely reflect the natural course and causative background of human NAFLD. Thus, it would be best to validate the identified DEGs in patients with hepatic steatosis. Furthermore, the dietary animal model used here did not manifest high serum TG levels, although pigs developed severe hypercholesterolemia and hepatic steatosis. The amount and type of dietary fat and diet duration may have influenced these effects. These issues should be addressed in future studies.

## Conclusions

In summary, we demonstrated here that testosterone deficiency may aggravate hepatic steatosis and hypercholesterolemia in pigs fed an HFC diet, and these effects were improved by testosterone treatment. Importantly, hepatic transcriptomic analysis revealed that increased hepatic steatosis induced by testosterone deficiency and an HFC diet was mediated by altered expression of genes involved in multiple metabolic processes. We observed abnormal regulation of fatty acid metabolism and found that the expression of fatty acid oxidation-related genes was reduced in the livers of CM pigs. Moreover, genes associated with immune and inflammatory responses, oxidative stress, and apoptosis also contributed to the increased hepatic steatosis. These observations provide a molecular basis for understanding the mechanisms through which testosterone deficiency aggravates the progression of diet-induced hepatic steatosis.

## Methods

### Experimental animals

Eighteen sexually mature male Chinese Wuzhishan (WZS) miniature pigs (6–7 months old) were obtained from the Institute of Animal Sciences, Hainan Academy of Agricultural Sciences (Haikou, China). The animals were housed in single pens with a 12-h light/dark cycle. The room temperature was maintained at 22 ± 3°C, with a relative air humidity of 50% ± 20%. Before and after their arrival, the animals were fed a standard swine diet and had free access to water. The animals received a standard diet without cholesterol during a 7-week “pretreatment period” for acclimation to the environment and baseline determinations. At week 7, the pigs were either surgical castrated or given a sham operation as described previously [81]. Testosterone was administrated weekly to castrated pigs via intramuscular injection with testosterone propionate (10 mg/kg body weight; Sigma-Aldrich, St. Louis, MO, USA) dissolved in corn oil [82]. Testosterone replacement therapy was given on the same day of castration to avoid disruption of hormonal influences. The pigs were fed an HFC diet starting from week 8 and were divided into three groups (n = 6 animals/group) as follows: intact male pigs fed an HFC diet (IM), castrated male pigs fed an HFC diet (CM), and castrated pigs with testosterone replacement fed an HFC diet (CMT). The HFC diet was comprised of 73% normal swine diet, 15% lard, 10% egg yolk power, 1.5% cholesterol, and 0.5% sodium cholate. The HFC diet was similar to an atherogenic diet, which has been shown to induce hepatic steatosis and atherosclerosis [38,83]. Body weights were recorded every week for 12 weeks. All experimental procedures used in this study were approved by the Institutional Animal Care and Use Committee of the Zhejiang Chinese Medical University (Hangzhou, China).

At the end of the experimental period, the animals were killed by exsanguination under sodium pentobarbital anesthesia. The carcasses were eviscerated according to the procedures as previously described [19], and the carcass fat from the left side of each animal was weighed. Livers were removed and weighed, and liver weight indexes were calculated as the liver weight/body weight ratio (g/kg). Livers were then frozen immediately in liquid nitrogen and stored at −80°C for further analysis.

### Serum measurements

Fasting blood samples were collected prior to castration and twice weekly throughout the study. Sera were separated from collected blood samples by centrifugation at 3000 × *g* at 4°C for 15 min and stored at −80°C for further analysis. Serum testosterone and Insulin concentrations were measured at week 7 (0 w; the start of the experimental period after the 7-week acclimation) and week 19 (12 w; the end of the experimental period), using a commercial RIA kit (Beijing North Institute of Biological Technology, Beijing, China). Serum TGs, TC, HDL-C, LDL-C, glucose, ALT, and AST were measured with an Automatic Biochemistry Analyzer (Hitachi 7020, Tokyo, Japan). HOMA-IR was calculated using the formula, base glucose × base insulin/22.5 [8,26]. Serum FFAs, SOD, glutathione peroxidase (GSH-PX), and MDA were measured using commercially available kits (Nanjing Jiancheng Biotech, Inc., Nanjing, China).

### Biochemical analysis in liver samples

Hepatic lipids were measured using the methods described by Shi et al. [84] with slight modifications. Briefly, liver samples from each pig were homogenized at 4°C in phosphate-buffered saline (PBS, pH 7.2). Supernatants were then centrifuged at 3000 × *g* for 10 min at 4°C, and TG and TC levels were determined using commercially available kits (Rongsheng Biotech, Inc., Shanghai, China) according to the manufacturer’s instructions. Protein concentrations in the liver samples were measured with a BCA Assay Kit (Pierce, Rockford, IL, USA).

### Liver histology

For histopathological analysis, pig liver tissues were fixed in 10% formalin, embedded in paraffin, and sectioned at 5-μm thicknesses using a microtome (Leica, Wetzlar, Germany). Subsequently, sections were stained with hematoxylin and eosin (H&E). In addition, frozen liver samples were embedded in Tissue-Tek OCT Compound (Sakura Finetek, Torrance, CA, USA), sectioned at 10-μm thicknesses using a cryostat (Leica), and stained with Oil Red O (Sigma-Aldrich) to study fat deposition. For each of the indicated histological procedures, representative photomicrographs were taken with a light microscope (Nikon Eclipse 80i, Nikon, Tokyo, Japan).

### cDNA library preparation and Illumina sequencing

Liver tissues from pigs were subjected to total RNA extraction using TRIzol reagent (Invitrogen, Carlsbad, CA, USA) according to the manufacturer’s instructions. RNA samples were purified using an RNeasy Mini Kit (Qiagen, Hilden, Germany). RNA integrities were assessed with a 2100 Bioanalyzer (Agilent Technologies, Inc., Santa Clara, CA, USA) and agarose gel electrophoresis. All samples had a RNA integrity number (RIN) of more than 8.5. To reduce variation among individuals within each of the three groups, total RNA from pigs of the same group was pooled together in equal amounts to generate a mixed sample [85,86]. These three pooled RNA samples were subsequently used for cDNA library construction and Illumina deep sequencing. Sequencing libraries were prepared using a TruSeq RNA Sample Preparation Kit (Illumina, San Diego, CA, USA) according to the manufacturer’s instructions. Briefly, 10 μg of each total RNA sample was processed via poly-A selection with oligo(dT) magnetic beads and fragmentation. The resulting fragmented mRNAs were then processed by first-strand cDNA synthesis using reverse transcription with random primers, followed by second-strand cDNA synthesis using DNA polymerase I and RNase H (Invitrogen). Paired-end (PE) oligo adapters (Illumina) were then added to the cDNA fragments with T4 ligase. The resulting cDNA fragments were purified and enriched by polymerase chain reaction (PCR). The cDNA libraries were sequenced with an Illumina HiSeq2000 (Illumina), which generated paired-end raw reads of approximately 100-bp in size.

Raw sequencing data were evaluated by FAST-QC (http://www.bioinformatics.babraham.ac.uk/projects/fastqc), an online bioinformatics program used to characterize quality distributions of nucleotides, position-specific sequencing qualities, GC contents, the proportions of PCR duplication, and k-mer frequencies [87]. Raw reads after quality control testing were then mapped to the reference pig genome version 10.2 (Sscrofa 10.2), using TopHat software [88] with default parameters.

### Analysis of DEGs

We applied the DEGSeq [22] algorithm to filter DEGs. The resulting significance scores were corrected for multiple testing using the Benjamini-Hochberg (BH) method [23]. An FDR of 0.05 or less and an absolute fold change of 1.5 or more were set as thresholds to evaluate the significance of gene expression differences [24]. The clustering software Cluster 3.0 was used to perform hierarchical clustering analysis. Clustering results were visualized using the Java TreeView program [89].

### Functional enrichment analysis

To investigate gene functions and uncover signaling networks/pathways among the selected DEGs, GO analysis was applied to analyze the main function of DEGs according to the NCBI Gene Ontology database, which provides key functional classifications for genes [90]. According to the methods described by Wang et al. [91], Fisher’s exact test and the *χ*^2^ test were used to classify the GO terms, and the FDR was calculated to correct the *P*-value. The smaller the FDR, the smaller the error would be when evaluating the *P*-value. The FDR was defined as $DR=1-\frac{N_k}{T}$, where *N*_*k*_ refers to the number of Fisher’s test *P*-values less than the *χ*^2^ test *P*-values. Enrichment provided a measure of the significance of the function, such that as the enrichment was increased, the corresponding function was more specific, which aided in experimentally identifying GOs with more concrete functions [91]. GO categories with *P*-values of less than 0.01 after correction by FDR were selected for further analysis.

Pathway analysis was used to determine significant pathways associated with DEGs according to KEGG (http://www.genome.jp/kegg). As with the GO analysis, Fisher’s exact test was used to classify significantly enriched pathways, and the resulting *P*-values were adjusted using the FDR algorithm. During the analysis of KEGG pathway terms, we required corrected *P*-values to be less than 0.05. In addition, the enriched pathways were selected to build Pathway-Act-Networks according to the relationships identified between the pathways in the KEGG database. Pathway assignments were also performed using the KEGG database and KegArray software [92].

The expression profiles of DEGs were determined by cluster analysis based on the STEM method (http://www.cs.cmu.edu/~jernst/st/) [25,93]. Significant profiles were identified using Fisher’s exact test and multiple comparisons. GO analysis was also applied to the genes belonging to specific profiles.

### qRT-PCR analyses

Total RNA from liver specimens was isolated using TRIzol reagent (Invitrogen) according to the manufacturer’s instructions. Approximately 1 μg total RNA was used for first-strand cDNA synthesis, which was carried out using an MMLV-RT Kit (Promega, Madison, WI, USA) according to the manufacturer’s protocol. qRT-PCR was performed using the StepOnePlus Real-Time PCR Detection System (Applied Biosystems, Inc., Foster City, CA, USA). The primers used for measuring the expression of mRNAs of interest are listed in Additional file 14. The amplifications were performed in 20-μL reaction mixtures containing 10.4 μL of 2× SYBR Premix Ex Taq (TaKaRa, Dalian, China), 0.4 μL of each primer (10 mM), 7.8 μL distilled water, and 1.0 μL cDNA. The following PCR conditions were used for all genes: 95°C for 5 min, followed by 40 cycles of 95°C for 15 s, 60°C for 30 s, and 72°C for 30 s. After amplification, melt curve analysis was performed to confirm the specificity of the reaction. All measurements were performed in triplicate. β-Actin was used as a reference gene to normalize gene expression. The 2^-∆∆CT^ method was used to analyze the qRT-PCR data and assign relative expression differences [94].

### Immunohistochemistry analysis

Immunohistochemistry was performed with paraffin-embedded sections using standard protocols. Briefly, liver sections were deparaffinized in xylene and rehydrated through a graded ethanol series. Endogenous peroxidase was blocked with 3% hydrogen peroxide (H_2_O_2_). The sections were boiled in 10 mM citrate buffer (pH 6.0) for 15 min for antigen retrieval. After blocking with 5% normal goat serum, sections were incubated at 4°C overnight with the following primary antibodies: anti-CCL2 (1:100; ab7814, Abcam, Cambridge, MA, USA), anti-CXCL9 (1:50; bs-2551R, Bioss, Beijing, China), and anti-IRF7 (1:250; bs-2994R, Bioss). Thereafter, the sections were rinsed in PBS and incubated with a biotinylated secondary antibody. The antigen was visualized with a diaminobenzidine (DAB) kit (Vector Laboratories, Burlingame, CA, USA) and counterstained with hematoxylin. A negative control without the primary antibody was included. Images were acquired using a Nikon Eclipse 80i fluorescence microscope and a DS-Fil CCD camera (Nikon).

### Western blot analysis

Total protein was extracted from frozen liver samples (~80 mg) as described previously [2]. Samples were centrifuged for 15 min at 4°C and 12,000 × *g*, and supernatants were collected. Protein concentrations were measured with a BCA Protein Assay Kit (Pierce). After denaturation, liver protein samples were resolved by 10% sodium dodecyl sulfate-polyacrylamide gel electrophoresis and transferred to polyvinylidene membranes (Millipore Corp., Billerica, MA, USA). Membranes were stained with Ponceau S to visually check the quality of proteins that were transferred and were then blocked for 1 h in 1× TBS with 5% nonfat milk. Primary antibodies against CPT1A (1:400; SAB2100476, Sigma-Aldrich), PPARD (1:600; AV32878, Sigma-Aldrich), and GAPDH (1:600; SC-16654, Santa Cruz Biotechnology, Santa Cruz, CA, USA) were incubated with the membrane over night at 4°C. Membranes were then washed and incubated with Odyssey Infrared-labeled secondary antibodies (LI-COR Biosciences, Lincoln, NE, USA) for 1 h in the dark at 37°C. After the last washing step, the membranes were scanned and analyzed using an Odyssey Infrared Imaging System (LI-COR Biosciences).

### Statistical analysis

Statistical analysis was performed using SPSS 13.0 software (SPSS, Chicago, IL, USA). The results are presented as means ± SEMs. Statistical differences between groups were examined using the unpaired Student’s *t*- test. Differences with *P*-values of less than 0.05 were considered statistically significant.

### Data accessibility

The sequence data from this study have been submitted to the Gene Expression Omnibus (GEO) database (http://www.ncbi.nlm.nih.gov/geo) under accession number GSE65696.

## Additional files

Additional file 1:
**The list of common genes expressed IM, CM and CMT pigs.**
Additional file 2:
**Lists of differentially expressed genes (DEGs) between groups of pigs.** **Table S1.** lists of DEGs between CM and IM; **Table S2.** lists of DEGs between CMT and CM; **Table S3.** lists of DEGs between CMT and IM. **Table S4.** lists of DEGs between any two groups. Additional file 3:
**GO enrichment analysis of differentially expressed genes (DEGs) between CM and IM.**
Additional file 4:
**GO enrichment analysis of differentially expressed genes (DEGs) between CMT and CM.**
Additional file 5:
**GO enrichment analysis of all significant differentially expressed genes (DEGs) between any groups of pigs.**
Additional file 6:
**Pathway analysis of all significant differentially expressed genes (DEGs) between any groups of pigs.**
Additional file 7:
**The expression patterns of all significant differentially expressed genes (DEGs) between any groups of pigs by model profiles.** Figure showing the expression patterns of 2595 genes were summarized by 7 model profiles. Each box represents a model profile. The upper number in the profile box is the model profile number and the *p*-value is shown. Two expression patterns of genes had significant *p*-values (*p* <0.05) (green and red colored boxes). Additional file 8:
**The genes involving significant profiles.** Table lists the differentially expressed genes (DEGs) assigned in each significant expression profile. Additional file 9:
**Significant enriched GO terms and contributing genes in profile 5.**
Additional file 10:
**Significant enriched GO terms and contributing genes in profile 2.**
Additional file 11:
**The pathways used for pathway-net analysis.** **Table S1.** The pathways list of pathway-net analysis; **Table S2.** The power of the interaction among pathways. Additional file 12:
**Differences in hepatic gene expression in the KEGG fatty acid degradation pathway.** (A) The CM group compared to the IM group. (B) The CMT group compared to the CM group. The colored small boxes correspond with genes detected in this study. Green: downregulated expression; red: upregulated expression; gray: unchanged expression. The numbers within the small boxes are enzyme codes. IM: intact male pigs fed an HFC diet; CM: castrated male pigs fed an HFC diet; CMT: castrated male pigs fed an HFC diet and given testosterone replacement therapy. Additional file 13:
**Differences in hepatic gene expression in the KEGG peroxisome pathway.** (A) The CM group compared to the IM group. (B) The CMT group compared to the CM group. The colored small boxes correspond with genes detected in this study. Green: downregulated expression; red: upregulated expression; gray: unchanged expression. IM: intact male pigs fed an HFC diet; CM: castrated male pigs fed an HFC diet; CMT: castrated male pigs fed an HFC diet and given testosterone replacement therapy. Additional file 14:
**Information on primers used for quantitative real-time RT-PCR.**

## Abbreviations

ALT: Alanine aminotransferase
ANOVA: Analysis of variance
AR: Androgen receptor
AST: Aspartate aminotransferase
DAB: Diaminobenzidene
DEG: Differentially expressed gene
FDR: False discovery rate
FFA: Free fatty acid
GO: Gene ontology
GSH-PX: Glutathione peroxidase
H&E: Hematoxylin and eosin
HDL-C: High-density lipoprotein cholesterol
HFC: High-fat and high-cholesterol
LDL-C: Low-density lipoprotein cholesterol
NAFLD: Nonalcoholic fatty liver disease
NASH: Nonalcoholic steatohepatitis
PBS: Phosphate-buffered saline
PE: Paired-end
qRT-PCR: Quantitative real-time reverse transcription polymerase chain reaction
RIN: RNA integrity number
RNA-Seq: RNA sequencing
SEM: Standard error of the mean
SOD: Superoxide dismutase
T: Testosterone
TC: Total cholesterol
Tfm: Testicular feminized mice
TG: Triglyceride
STEM: Short Time-Series Expression Miner
