# Correction to: Transcriptomic analysis of hepatic responses to testosterone deficiency in miniature pigs fed a high-cholesterol diet

**DOI:** 10.1186/s12864-020-6485-4

**Published:** 2020-01-16

**Authors:** Zhaowei Cai, Xiaoling Jiang, Yongming Pan, Liang Chen, Lifan Zhang, Keyan Zhu, Yueqin Cai, Yun Ling, Fangming Chen, Xiaoping Xu, Minli Chen

**Affiliations:** 1 Laboratory Animal Research Center, Zhejiang Chinese Medical University, Hangzhou, 310053 China; 2 Department of Cancer Genetics, Roswell Park Cancer Institute, Elm and Carlton Streets, Buffalo, NY 14263 USA; 3 College of Animal Science, Nanjing Agricultural University, Nanjing, 310058 China

**Correction to: BMC Genomics**

**https://doi.org/10.1186/s12864-015-1283-0**

Following the publication of the original article [1], it was reported that the accession number given in the ‘Data accessibility’ declaration, GSE65696, is incorrect. The correct number is GSE62696.
