# Supplementary figures and images for: Transcriptomic analysis of hepatic responses to testosterone deficiency in miniature pigs fed a high-cholesterol diet
Source: BMC Genomics. 2015 Feb 6;16(1):59. doi: 10.1186/s12864-015-1283-0 (PMC4328429; doi:10.1186/s12864-015-1283-0)

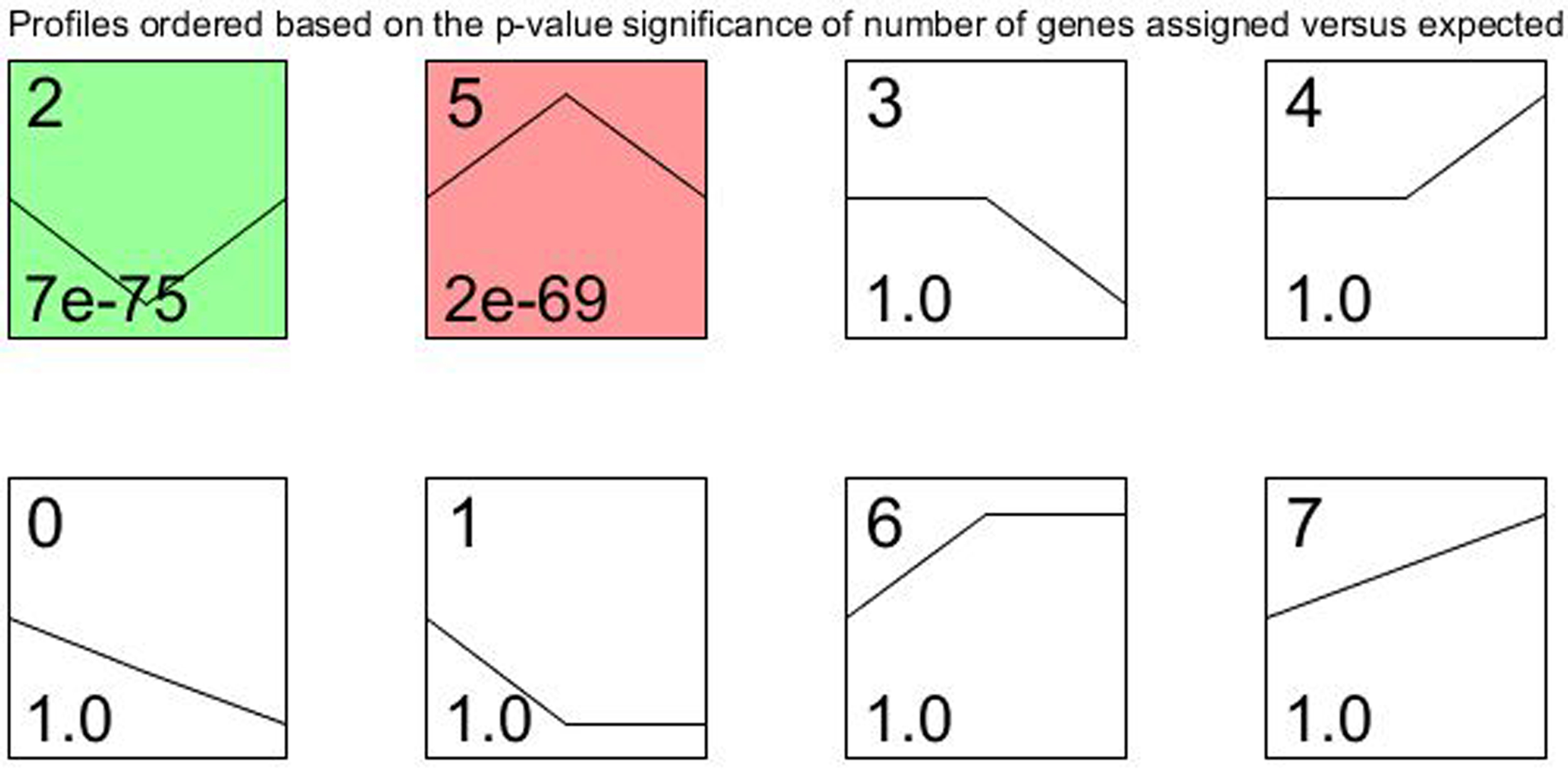

Supplement: Additional file 7: — The expression patterns of all significant differentially expressed genes (DEGs) between any groups of pigs by model profiles. Figure showing the expression patterns of 2595 genes were summarized by 7 model profiles. Each box represents a model profile. The upper number in the profile box is the model profile number and the p-value is shown. Two expression patterns of genes had significant p-values (p <0.05) (green and red colored boxes). [file 12864_2015_1283_MOESM7_ESM.tiff]

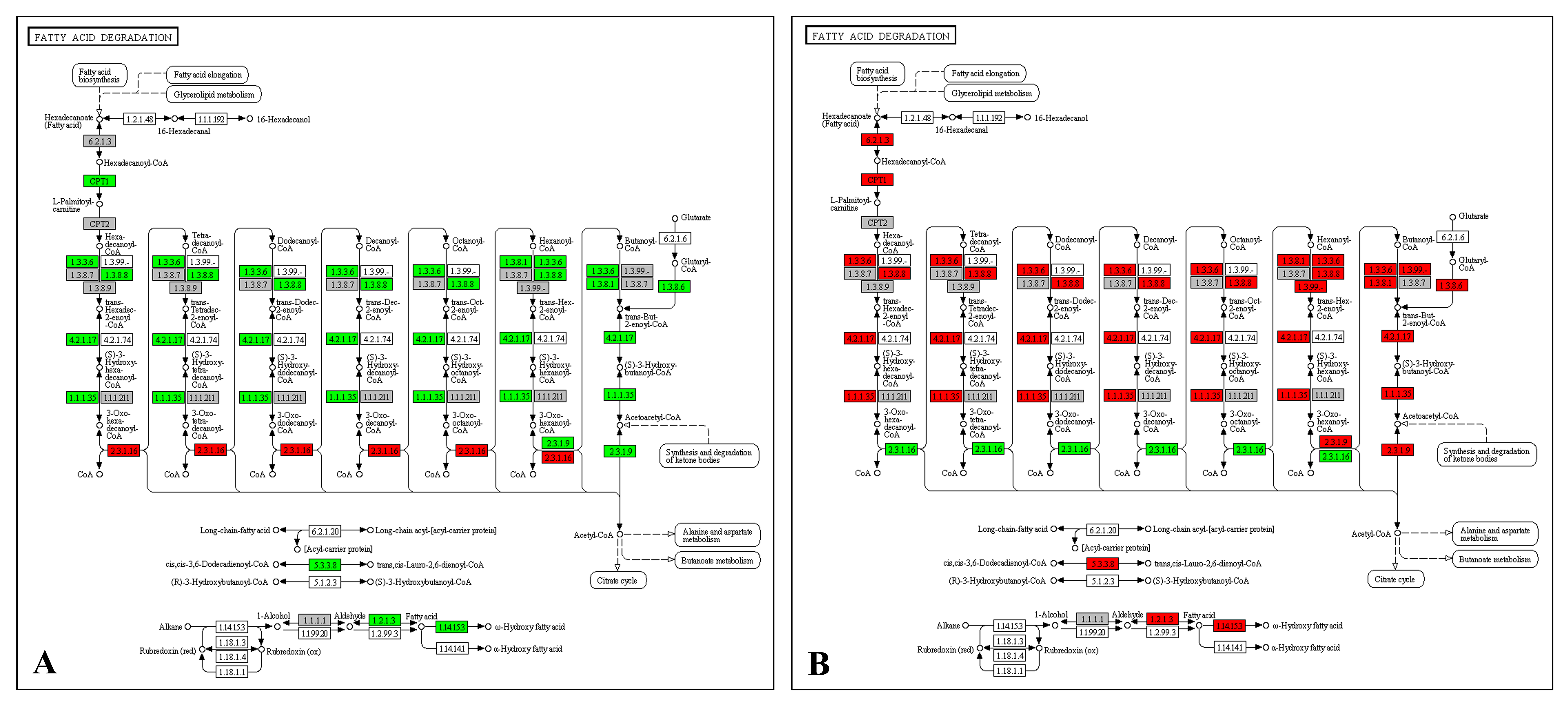

Supplement: Additional file 12: — Differences in hepatic gene expression in the KEGG fatty acid degradation pathway. (A) The CM group compared to the IM group. (B) The CMT group compared to the CM group. The colored small boxes correspond with genes detected in this study. Green: downregulated expression; red: upregulated expression; gray: unchanged expression. The numbers within the small boxes are enzyme codes. IM: intact male pigs fed an HFC diet; CM: castrated male pigs fed an HFC diet; CMT: castrated male pigs fed an HFC diet and given testosterone replacement therapy. [file 12864_2015_1283_MOESM12_ESM.tiff]

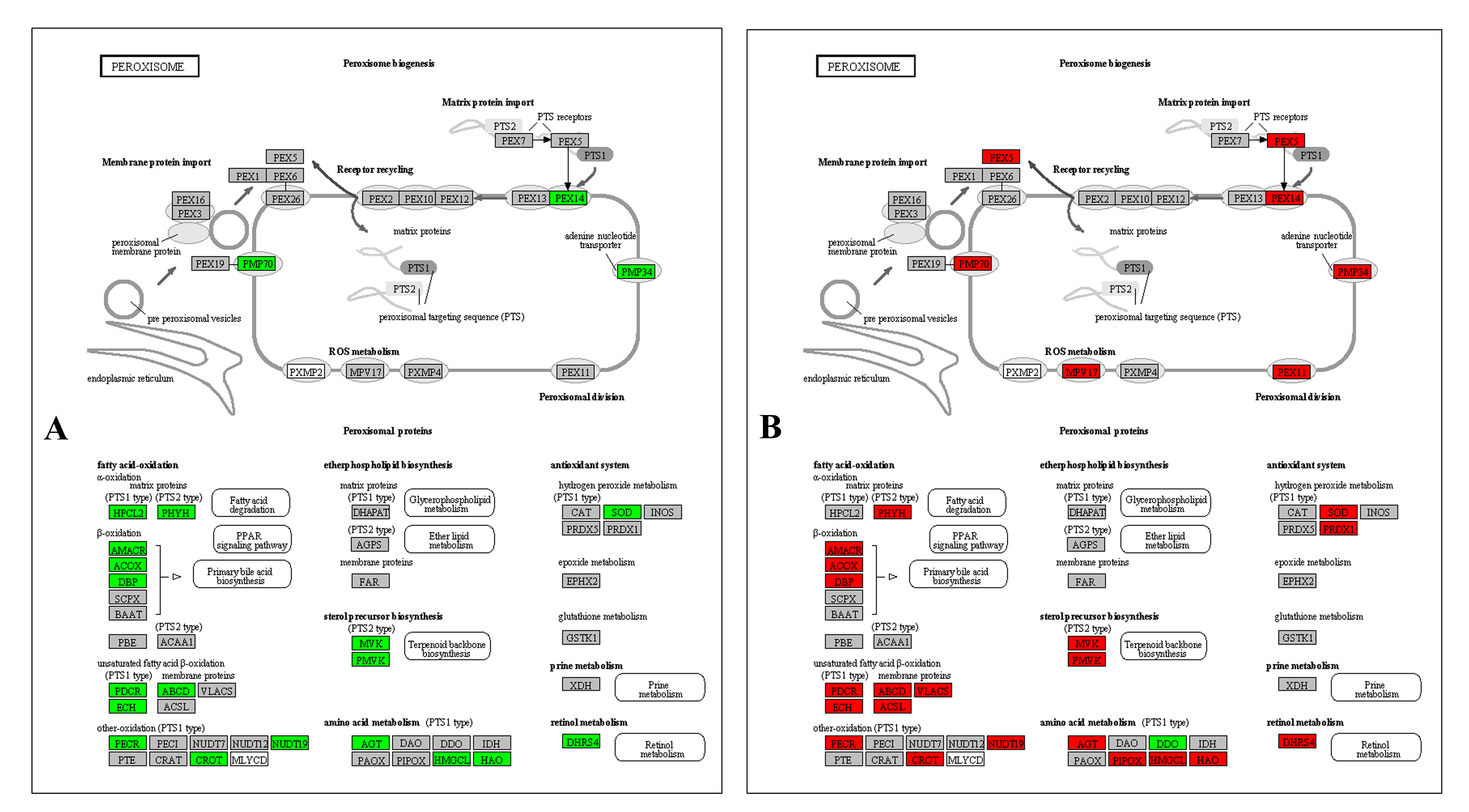

Supplement: Additional file 13: — Differences in hepatic gene expression in the KEGG peroxisome pathway. (A) The CM group compared to the IM group. (B) The CMT group compared to the CM group. The colored small boxes correspond with genes detected in this study. Green: downregulated expression; red: upregulated expression; gray: unchanged expression. IM: intact male pigs fed an HFC diet; CM: castrated male pigs fed an HFC diet; CMT: castrated male pigs fed an HFC diet and given testosterone replacement therapy. [file 12864_2015_1283_MOESM13_ESM.tiff]
